# Diagnostic challenges in infected cystic papillary renal cell carcinoma following recent right limited hemicolectomy

**DOI:** 10.1093/jscr/rjaf571

**Published:** 2025-07-25

**Authors:** Hamzeh Farraj, Abdullah A Al-Saa'd

**Affiliations:** Department of Specialized Surgery, Faculty of Medicine, Al-Balqa Applied University, Sixty street Salt, Balqa, 19117, Jordan; Urology Section, Surgery Department, Islamic Hospital, King Hussein street, Al Abdali Amman 11181, Jordan

**Keywords:** rare diseases, kidney, infections, renal cell carcinoma

## Abstract

In this case we present a diagnostic challenge of a renal cyst that has radiological features that were hard to differentiate between infection and malignancy. Our patient presented with suspicious infected right renal cystic lesion, 4 months after undergoing complicated appendectomy with limited right hemicolectomy. This cyst was diagnosed previously as Bosniak IIF and was assigned for follow up every 6 months. Radiological assessment was in favor of abscess formation however it was also concerning for infected renal cell cancer. As only drainage may cause seeding of potential cancer cells, right partial nephrectomy was done. Histopathology was papillary cancer. Bosniak IIF cystic lesions are still managed with follow up, however this type of cystic lesions carries considerable risk of malignancy according to most recent studies; we are presenting this case report to emphasis this fact and to highlight the importance of proper diagnosis and treatment choice.

## Introduction

Renal cell cancer (RCC) is the most common renal neoplasm that originates from the renal cortex [1]. It is commonly found as an incidental solid renal mass on imaging modalities or as a BosniakIII or IV renal cyst. However, it may come with flank pain, hematuria or other constitutional symptoms [2]. Risk factors for RCC include smoking, obesity, familial history, end stage renal disease and hypertension [3]. The best imaging modality to diagnose RCC is computed tomography (CT) [4]. In this report we present a case of potentially infected right BosniakIIF renal cyst. Those cysts carry a risk to progress to malignancy or may harbor malignant cells and need close follow up. However, our patient presented with acute illness that mandates intervention which would be only aspiration if no cancer risk was existing. Our aim is to highlight the management approaches of infected complex renal cyst with diagnostic challenge.

## Case presentation

**Figure 1:**
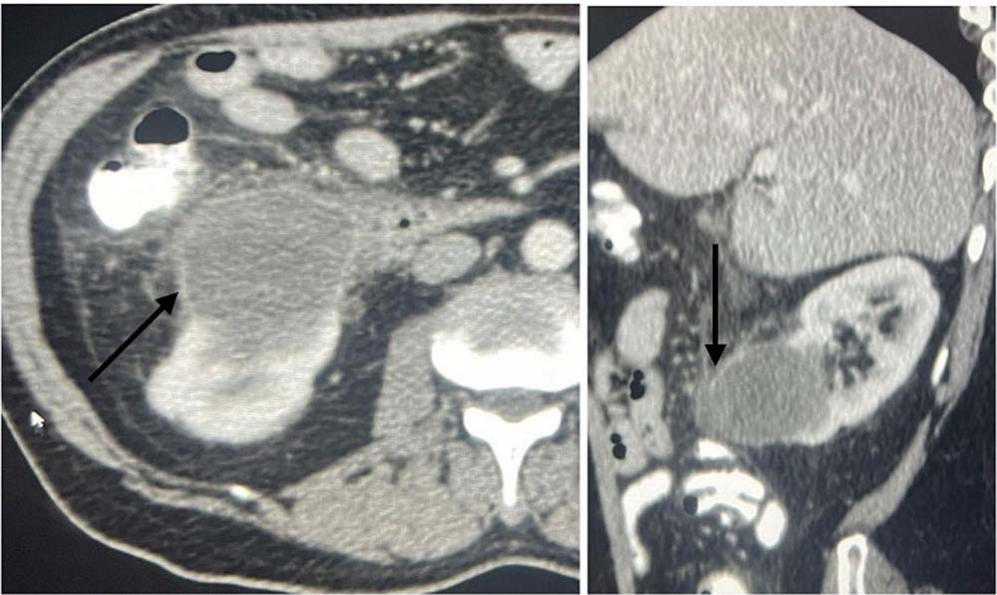
An axial view (on the right) and sagittal view (on the left) showing a ring enhancing lower pole cyst (arrow), suggesting infected cyst vs abscess formation.

**Figure 2:**
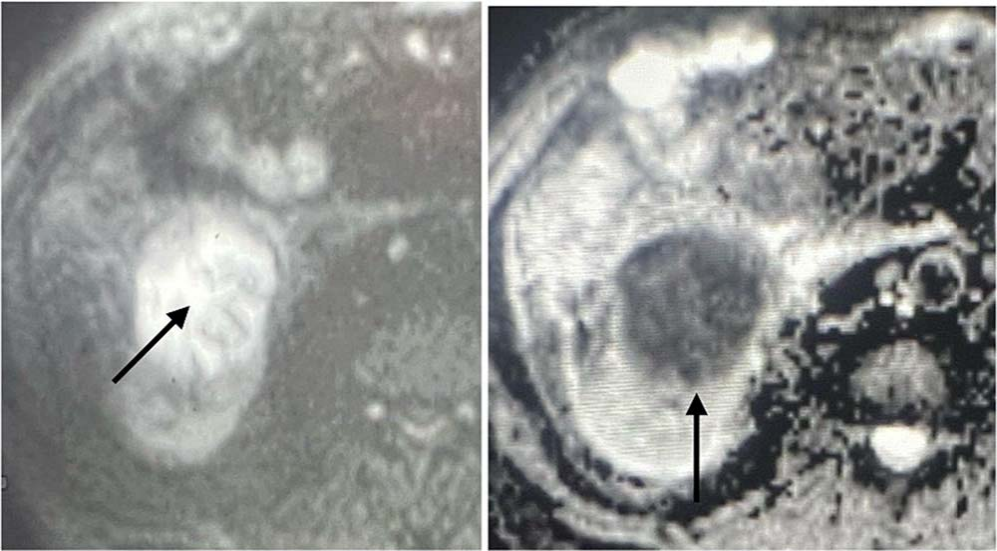
DWI- ADC map shows strong internal restricted diffusion which can present in both abscess as well as tumor (arrow).

**Figure 3:**
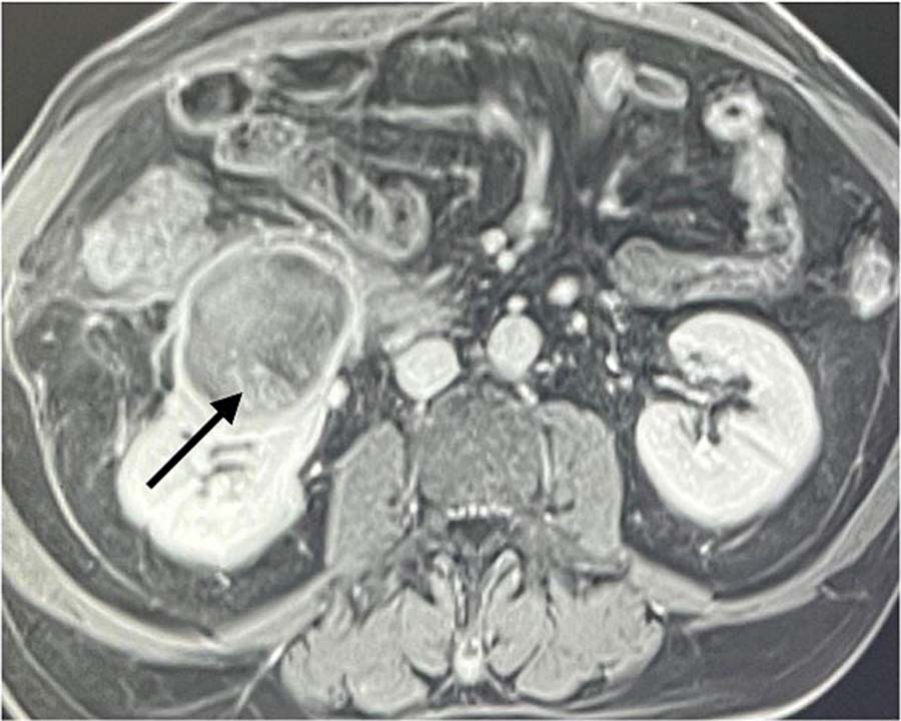
T1 post contrast showing a ring enhancing cystic lesion in the lower pole with fine internal enhancement (arrow).

A 60-year-old male patient was referred to our clinic due to concern of infected renal cyst. Five months prior to referral, he underwent an open appendectomy and right limited hemicolectomy due to perforated appendix. At that time he was found to have accidental right Bosniak IIF renal cyst on CT scan and follow up every 6 months was recommended by urologist. Four months later, he came to emergency department with fever, chills and right flank pain. CT scan of the abdomen and pelvis with intravenous contrast was done and showed same size exophytic cyst in the lower portion of the right kidney measuring 4.9 × 5.2 cm but with new finding of rim enhancement with surrounding fat stranding suggesting complicated cyst with possible abscess formation. There was fat stranding and wall thickening of the adjacent ascending colon (Fig. 1). These findings couldn’t differentiate between infected cyst and cancer. Due to the high risk of malignancy associated with Bosniak IIF renal cyst and the challenging differential diagnosis, we further discussed the case with radiologist and decision was made to do an magnetic resonance imaging (MRI) with contrast for further evaluation and for better visualization of internal enhancement. Most MRI findings were suggestive of infected cyst. However, diffusion-weighted imaging and apparent diffusion coefficient (DWI-ADC) map showed strong internal restricted diffusion which is not specific to either abscess or tumor. Reaching the diagnosis based solely on these initial imaging findings was challenging. T1 post contrast image revealed fine internal enhancement suggesting more complex lesion than a simple infected renal cyst or abscess, raising suspicion for a neoplastic process (Figs 2 and 3).

**Figure 4:**
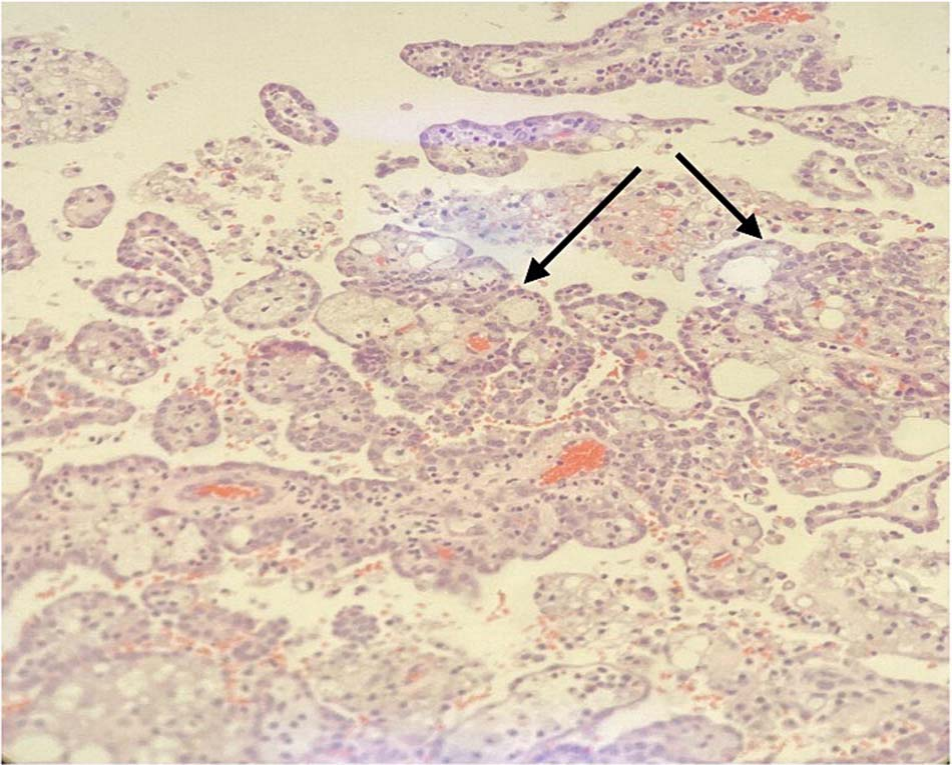
Papillary structures with fibrovascular core (arrow) lined by low grade tumor cells. H&E stain, 100×.

**Figure 5:**
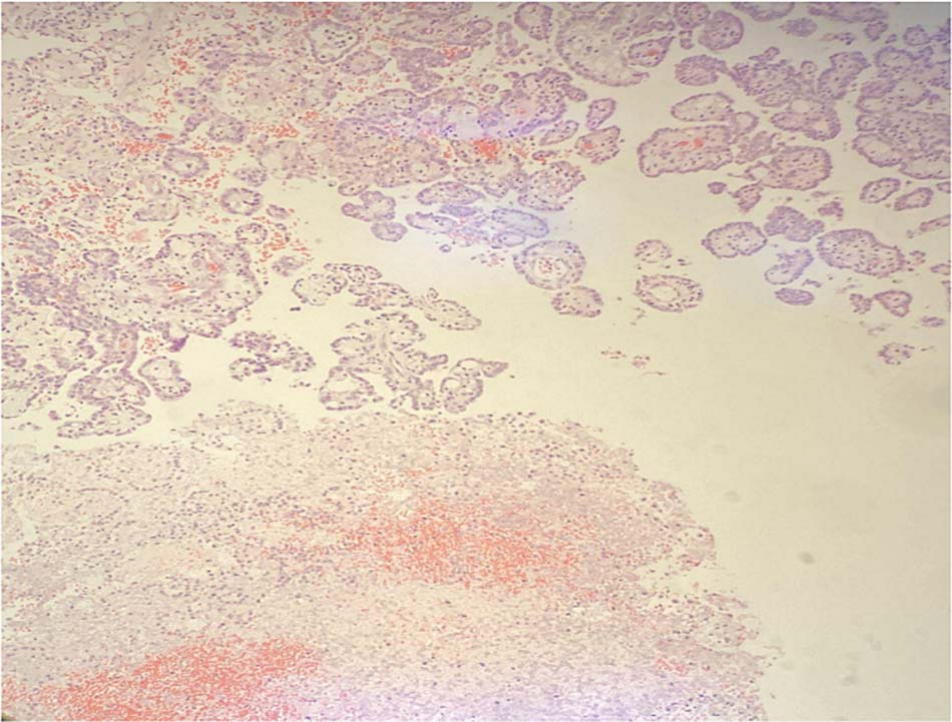
Diffuse tumor necrosis in the lower field. H&E stain 40×.

We counseled the patient regarding management options including percutaneous drainage versus surgical excision. The decision was made to proceed for surgical exploration to avoid risk of dissemination in the case of underling malignancy. The patient was prepared for surgery, a cross match was done, and two packed red blood cells were prepared. Informed consent was obtained for open cyst excision, with the possibility of conversion to partial or radical nephrectomy based on intraoperative findings and frozen section. During operation the cyst was well vascularized and firm in texture. It was excised and sent as a frozen section. It showed suspicious features of malignancy. Accordingly, partial nephrectomy was completed with a surgical margin, drain was inserted, and abdominal layers were closed. The drain was removed the following day. The patient was sent home on day 2 post op. Pathology was consistent with type 1 papillary RCC with a negative margin of 1.5 mm away from the tumor (Figs 4 and 5).

## Discussion

Cystic renal masses are classified according to the Bosniak classification system. According to a recent meta-analysis and systematic review study, the proportion of malignancy according to version 2019 Bosniak classification in class IIF cystic masses is 46% [5]. Bosniak IIF cysts need careful follow-up due to higher risk of malignancy those cysts may harbor. Follow-up every 6 months in the first year and then yearly is recommended [5].

RCC can be presented with uncommon symptoms. RCC rarely present with gastrointestinal symptoms and liver metastasis. Utkarsh Ojha reported a case of a 49-year-old man who presented with upper gastrointestinal symptoms including nausea, heartburn, and weight loss. Physical exam was significant for hepatomegaly and blood testing was significant for raised C-reactive protein and alkaline phosphatase. According to that, patient was referred to GI. Eventually, CT scan revealed lung and liver spread and large left upper pole renal cancer with left renal vein thrombus [6]. The patient in our case was known to have premalignant cyst and was on regular follow up until he presented with infection like symptoms that mandate drainage of the cyst. However, this management was not appropriate with the existing high potential of malignancy.

RCC can occasionally be presented as renal abscess or an infected complex cyst. While reviewing the literature, a case report showed a sarcomatoid RCC masquerading as a renal abscess in a patient presenting with fever and chills, not responsive to initial antibiotics. Unexpectedly, nephrostomy drainage fluid histology showed cancerous cells. Accordingly, a radical nephrectomy was done [7]. In that patient it would have been better if they could recognize the possibility of cancer radiologically and avoid aspiration and potential tumor dissemination.

RCC can coexist with severe infection; a case report describes a patient presented with recurrent fever and flank pain as a case of emphysematous pyelonephritis with multiple abscesses and a staghorn stone. His preoperative CT scan was not in favor of underlying cancer. A laparoscopic right nephrectomy was done. Final histopathology analysis proved sarcomatoid squamous cell carcinoma of the renal pelvis with superimposed renal infection [8].

Our conclusion is that malignancy should always be suspected when we deal with Bosniak IIF cystic lesions when they present with acute symptoms even if the imaging changes were suggestive of abscess formation or infection. Aspiration or just drainage of infected complex renal cyst may impact patient prognosis and lead to cancer upstaging if the risk of malignancy is not addressed appropriately. Bosniak IIF renal cyst management need more study as the evidence for its high malignant risk is growing.
